# Supplementary material for: Enhancing Electrochemical Performance with g-C3N4/CeO2 Binary Electrode Material
Source: Molecules. 2023 Mar 8;28(6):2489. doi: 10.3390/molecules28062489 (PMC10059818; doi:10.3390/molecules28062489)
Supplement: Supplementary file 1 [file molecules-28-02489-s001.zip › molecules-2245560-supplementary.pdf]

SI file

1.1. Characterization

The structural features of as-prepared materials were analyzed by powder X-ray diffraction (X-ray diffractometer model XRD-6100, Shimadzu, Japan) with CuKα X-ray radiation ($\lambda = 0.15406$ nm). The morphological features were examined by the scanning electron microscopy (FESEM, Hitachi, S-4800 and HRTEM, Tecnai G2 F20 S-Twin at an accelerating voltage of 200 kV). The elements of active materials were recognized using energy dispersive X-ray spectroscopy (EDS) attached to the SEM. The chemical states of the materials were tested using a Thermo Scientific X-ray photoelectron spectroscopy (XPS) instrument utilizing Al Kα radiation ($\lambda = 1486.6$ eV).

1.2 Pre-treatment of Ni-foam

Foam nickel with the thickness of 2 mm was used as substrate. Before cleaning, the nickel foam was cut into rectangles of 15×5 mm^2. Then immerse the nickel foam in a solution of 20% hydrochloric acid solution and deionized water mixed in a ratio of 3:1 and wash in an ultrasonic cleaner for 14 min to remove the surface oxide. After that soak the nickel foam in deionized water and clean it in the ultrasonic cleaner for 14 min, replace with new deionized water when finished, ultrasonic clean for 1 min and repeat six times. Finally, replace the deionized water with ethanol and clean with the same procedure to remove the residual acid and organic matter from the surface. Dry in a vacuum oven at 90 °C for 12 h.

1.3. Electrochemical tests

The electrochemical activity of the electrodes was tested using a standard three-electrode cell, which consists of Hg/HgO and platinum mesh as the reference electrode and counter electrode. The working electrode was organized by mixing the active material, carbon black, and

polyvinylidene difluoride (PVDF) in a mass ratio of 8:1.5: 0.5 with N-methyl-2-pyrrolidone (NMP). This obtained slurry was then covered on nickel foam via the drop casting technique and dried in an oven at 90°C for 12 h. Cyclic voltammetry (CV), galvanostatic charge-discharge (GCD), and electrochemical impedance spectroscopy (EIS) were used to assess the electrochemical activity of the electrodes. The CV tests were carried out at several scan rates, ranging from 5 to 300 mV s^{-1} at a potential of 0.0 V to 0.6 V in a 1 mol L^{-1} KOH aqueous solution. The GCD tests were executed within the range of 0–0.5 V *v*s. Hg/HgO at various current densities. The electrochemical impedance spectroscopy (EIS) measurements were carried out in the frequency range from 100 Hz to 1 MHz at the open-circuit potential. All electrochemical experiments were performed using a Biologic SP-200 electrochemical workstation.

1.4. Preparation of gel electrolyte

In order to prepare the alkaline polyvinyl alcohol/potassium hydroxide (PVA/KOH) gel electrolyte, initially, 5.6 g of PVA was dissolved in 50 mL of pure deionized water at 90 °C the temperature with continuous vigorous stirring to get a clear solution. As a result, after 1 h we obtained a clear viscous solution. 6 g of KOH was liquefied in 10 mL deionized water, then dropped into the cleared PVA solution with continues stirring until complete dissolution and formation of a gel like solution, finally, PVA/KOH gel electrolyte cooled to room temperature for further use.

1.5. CeQDs/CN-3//AC device fabrication

A hybrid coin-cell-type asymmetric supercapacitor device (HCASDs) was developed with *in-situ* synthesized CeQDs/CN-3 nanostructure as positive electrode and active carbon and PVDF with Nafion (5 μL) in a mass ratio of 95:5 slurry was drop casted on a nickel foam to act as the negative electrode, separated with filter paper as separator. The CeQDs/CN-3 nanostructure was

estimated from weight change of the nickel foam before and after deposition. The specific capacitance (C_s) from charge –discharge curves in a three-electrode cell was intended using Eq. S1 [1-3]:

$$C_s = {(\Delta t)(I)}/{m\Delta V} \qquad \text{(S1)}$$

where I (mA) and t (s) is the discharge current and discharge time, ΔV (V) is the voltage drop upon discharging (apart from the IR drop), and m (mg) is the mass of the active material. In addition, the energy density (E_d) (Wh/kg) and power density (P_d) (W/ kg) of the device were estimated on the total mass of the active materials, as per Eqs. S2 and S3 [4–6]:

$$E_d = \frac{1}{2}\left[\frac{C_s\,(V_f - V_i)^2}{3.6}\right] \qquad \text{(S2)}$$

$$P_d = \frac{3600\ X\ E_d}{\Delta t} \qquad \text{(S3)}$$

where Δt and $(V_f - V_i)$ are discharge time (s) and potential window for discharge process (V), respectively.

Table: S1 A comparison of g-C_3N_4-based supercapacitor electrodes reports in previously published studied.

**Materials**	**Current density**	**Specific capacitance (F g^{-1})**	**Ref.**
MnO_2/g-C_3N_4	1 A g^{-1}	211	[7]
$NiCo_2O_4$/g-C_3N_4	1 A g^{-1}	325.7	[8]
$NiCo_2O_4$/MWCNT	1 A g^{-1}	374	[9]
PEDOT/g-C_3N_4	2 A g^{-1}	200	[10]
α-Fe_2O_3/g-C_3N_4	1 A g^{-1}	167	[11]
ZnS/g-C_3N_4	1 A g^{-1}	497.7	[12]
Tubular g-C_3N_4	1 A g^{-1}	233	[13]
Fe_3O_4/g-C_3N_4	1 A g^{-1}	56.7	[14]
$Ni(OH)_2$/g-C_3N_4	1 A g^{-1}	445.6	[15]
g-C_3N_4 nanofibers	1 A g^{-1}	263.8	[16]
g-C_3N_4	0.75 A g^{-1}	96.9	[This work]
g-C3N4/CeO_2QDs	0.75 A g^{-1}	202.5	[This work]

References:

[1] A.J. Bard, L.R. Faulkner, H.S. White, *Electrochemical Methods Fundamentals of Electrochemistry*, John Wiley & Sons (2001).

[2] L. Fang, Y. Qiu, T. Zhai, F. Wang, M. Lan, K. Huang, Q. Jing, Flower-like nanoarchitecture assembled from Bi_2S_3 nanorod/MoS_2 nanosheet heterostructures for high-performance supercapacitor electrodes, Colloids Surf. A Physicochem. Eng. Asp. 535 (2017) 41-48.

[3] H. Liu, H. Guo, N. Wu, W. Yao, R. Xue, M. Wang, W. Yang, Rational design of nickel-cobalt selenides derived from multivariate bimetal metal-organic frameworks for high-performance asymmetric supercapacitor, J. Alloys Compd. 856 (2021) 156535.

[4] S. Liu, K.S. Hui, K.N. Hui. Flower-like copper cobaltite nanosheets on graphite paper as high-performance supercapacitor electrodes and enzymeless glucose sensors. ACS Appl Mater Interfaces 8 (2016) 3258-3267.

[5] K.V. Sankar, R.K. Selvan, The ternary $MnFe_2O_4$/graphene/polyaniline hybrid composite as negative electrode for supercapacitors, J. Power Sources 275 (2015) 399-407.

[6] X. Bai, Q. Liu, H. Zhang, J. Liu, Z. Li, X. Jing, Nickel-cobalt layered double hydroxide nanowires on three-dimensional graphene nickel foam for high performance asymmetric supercapacitors, Electrochim. Acta 215 (2016) 492-499.

[7] Chang, X.; Zhai, X.; Sun, S.; Gu, D.; Dong, L.; Yin, Y.; Zhu, Y. MnO2/g-C3N4 nanocomposite with highly enhanced supercapacitor performance. Nanotechnology 2017, 28, 135705.

[8] Shan, Qian Yuan 1 ; Guan, Bo 1 ; Zhang, Jun Ming 1 ; Dong, Fan 2 ; Liu, Xiao Ying 1 ; Zhang, Yu Xin, Facile Fabrication of NiCo2O4@g-C3N4(C) Hybrids for High-Performance Supercapacitors, Journal of Nanoscience and Nanotechnology, Volume 19, Number 1, January 2019, pp. 73-80(8). https://doi.org/10.1166/jnn.2019.16441

[9] Pathak, M.; Jose, J. R.; Chakraborty, B.; Rout, C. S.; High Performance Supercapacitor Electrodes based on Spinel NiCo2O4@MWCNT Composite with Insights from Density Functional Theory Simulations. The Journal of Chemical Physics. 2020,152, 064706.

[10] Chen X, Zhu X, Xiao Y, Yang X. PEDOT/g-C3N4 binary electrode material for supercapacitors. J Electroanal Chem (Lausanne). 2015; 743:99–104.

[11] Li Xu a b, Jiexiang Xia b, Hui Xu c, Sheng Yin b, Kun Wang b, Liying Huang b, Leigang Wang a, Huaming Li, Reactable ionic liquid assisted solvothermal synthesis of graphite-

like C3N4 hybridized α-Fe2O3 hollow microspheres with enhanced supercapacitive performance, Journal of Power Sources Volume 245, 1 January 2014, Pages 866-874.

[12] Binbin Wei a , Hanfeng Liang b,∗ , Rongrong Wanga , Dongfang Zhanga , Zhengbing Qi c , Zhoucheng Wang, One-step synthesis of graphitic-C3N4/ZnS composites for enhanced supercapacitor performance, Journal of Energy Chemistry 27 (2018) 472–477.

[13] Muhammad Tahir,a Chuanbao Cao,*a Faheem K. Butt,a Faryal Idrees,a Nasir Mahmood,b Zulfiqar Ali,a Imran Aslam,a M. Tanveer,a Muhammad Rizwana and Tariq Mahmood, Tubular graphitic-C3N4: a prospective material for energy storage and green photocatalysis, J. Mater. Chem. A, 2013,1, 13949-13955.

[14] Y.Z. Wu a 1, M. Chen a 1, X.H. Yan a b, J. Ren a, Y. Dai a, J.J. Wang a, J.M. Pan a, Y.P. Wang a, X.N. Cheng, Hydrothermal synthesis of Fe3O4 nanorods/graphitic C3N4 composite with enhanced supercapacitive performance, Materials Letters Volume 198, 1 July 2017, Pages 114-117.

[15] Lei Shi a, Jinglin Zhang a, Huidi Liu a, Manni Que a, Xiang Cai b, Shaozao Tan a, Langhuan Huang, Flower-like Ni(OH)2 hybridized g-C3N4 for high-performance supercapacitor electrode material, Materials Letters Volume 145, 15 April 2015, Pages 150-153.

[16] Muhammad Tahir†, Chuanbao Cao*†, Nasir Mahmood‡, Faheem K. Butt†, Asif Mahmood‡, Faryal Idrees†, Sajad Hussain†, M. Tanveer†, Zulfiqar Ali†, and Imran Aslam, Multifunctional g-C3N4 Nanofibers: A Template-Free Fabrication and Enhanced Optical, Electrochemical, and Photocatalyst Properties, ACS Appl. Mater. Interfaces 2014, 6, 2, 1258–1265.
